# A 3-bp deletion of *WLS5* gene leads to weak growth and early leaf senescence in rice

**DOI:** 10.1186/s12284-019-0288-8

**Published:** 2019-04-29

**Authors:** Chunyan Zhao, Chaolei Liu, Yu Zhang, Yongtao Cui, Haitao Hu, Noushin Jahan, Yang Lv, Qian Qian, Longbiao Guo

**Affiliations:** State Key Laboratory of Rice Biology, China National Rice Research Institute, Hangzhou, 310006 China

**Keywords:** *wls5*, Weak growth, Leaf senescence, Rice

## Abstract

**Background:**

In rice (*Oryza sativa*) and other grains, weak growth (dwarfism, short panicle length, and low seed-setting rate) and early senescence lead to reduced yield. The molecular mechanisms behind these processes have been widely studied; however, the complex genetic regulatory networks controlling growth and senescence require further elucidation.

**Results:**

We isolated a mutant exhibiting weak growth throughout development and early senescence of leaf tips, and designated this mutant *weakness and leaf senescence5* (*wls5*). Histological analysis showed that the poor growth of *wls5* plants involved a reduction in cell length and number. Physiological analysis and transmission electron microscopy revealed that the *wls5* cells had abnormal chloroplasts, and the mutants underwent chlorophyll degradation triggered by accumulation of reactive oxygen species. Consistent with this, RNA sequencing revealed changes in senescence-related gene expression in *wls5* plants. The *wls5* mutants also exhibited significantly higher stomatal density and altered phytohormone contents compared with wild-type plants. Fine mapping delimited *WLS5* to a 29-kb region on chromosome 5. DNA sequencing of *wls5* identified a 3-bp deletion in the first exon of *LOC_Os05g04900*, resulting in a deletion of a lysine in the predicted protein. Knockout of *LOC_Os05g04900* in Nipponbare plants caused leaf senescence, confirming this locus as the causal gene for *WLS5*.

**Conclusions:**

We identified a novel mutant (*wls5*) that affects plant development and leaf senescence in rice. *LOC_Os05g04900*, encoding a protein of unknown function, is the causal gene for *wls5*. Further molecular study of *WLS5* will uncover the roles of this gene in plant growth and leaf senescence.

**Electronic supplementary material:**

The online version of this article (10.1186/s12284-019-0288-8) contains supplementary material, which is available to authorized users.

## Background

Abnormal development, such as weak growth (dwarf height and small tissues and organs) and early senescence, has major effects on rice yield (Sakamoto and Matsuoka 2008; Pan et al. 2013; Liu et al. 2016; Hong et al. 2018). Additionally, premature leaf senescence leads to low photosynthetic efficiency and decreased accumulation of photosynthetic assimilates in reproductive organs, thus decreasing yield (Mitchell and Sheehy 2006; Yang et al. 2016a; Mao et al. 2017). These two factors are regulated by genetics and often triggered by environmental stresses. Therefore, understanding the molecular mechanisms underlying growth and leaf senescence will benefit rice breeding and production.

Plant senescence is controlled by complex regulatory networks involving coordinated action at the cellular, tissue, organ and organism levels (Lim et al. 2007). Typical senescence symptoms include physiological alterations such as chlorophyll degradation, reactive oxygen species (ROS) scavenging, carbon and nitrogen imbalances, and hormone responses (Lim et al. 2007; Hong et al. 2018). Genes associated with leaf senescence in rice have been isolated and characterized (Leng et al. 2017a), including transcription factors, receptors and signaling components for hormones and stress responses, and metabolic regulators (Mitchell and Sheehy 2006; Yamada et al. 2014; Wu et al. 2016; Yang et al. 2016a; Yang et al. 2016b; Zhao et al. 2016; Deng et al. 2017; Leng et al. 2017b; Mao et al. 2017; Hong et al. 2018; Ke et al. 2019). Senescence of plants can be induced by many factors, including reproductive growth, phytohormones, and environmental cues (Lim et al. 2007); moreover, the senescence-associated genes regulated during early leaf senescence remain largely unknown.

Plant growth and development are driven by cell division and expansion, fundamental and dynamic cellular processes that enable plants and various organs to develop to suitable sizes (Duan et al. 2012). Orderly growth and development involve many genes and pathways that affect plant organ size by altering cell number, cell size, or both (Krizek 2009). Plant development and leaf senescence involve some of the same regulatory factors. Indeed, several early senescence mutants show accompanying defects in plant development (Wu et al. 2016; Xie et al. 2016; El et al. 2017; Leng et al. 2017b; He et al. 2018; Hong et al. 2018**)**. For instance, a point mutation in *O. sativa* nicotinate phosphoribosyltransferase (*OsNaPRT1*) leads to dwarfism and a withered leaf tip phenotype (Wu et al. 2016); *dwarf and early-senescence leaf1* (*del1*) mutants exhibit dwarfism and an early leaf senescence phenotype, with abnormal growth caused by a reduction in cell number (Leng et al. 2017b). Moreover, *premature leaf senescence 3* (*pls3*) mutants show a semi-dwarf phenotype, early leaf senescence, and an early heading date compared to wild type (Hong et al. 2018), and the *premature senescence leaf 85* (*psl85*) mutant shows a distinct dwarfism and premature leaf senescence phenotype (He et al. 2018). Despite in-depth study of these mutants, the linkage between development and senescence is still poorly understood in rice.

In this study, we identified a mutant displaying abnormal plant growth and novel premature leaf senescence, designated *weakness and leaf senescence5* (*wls5*). Mutation of *WLS5* caused decreases in cell length, perturbed chloroplast development, and disturbed hormonal balance, resulting plants with fewer cells, increased ROS activity, and altered expression of senescence-associated genes. Our findings thus suggest that *WLS5* is a critical gene for plant growth and leaf senescence in rice.

## Main text

### Results

#### *wls5* mutants exhibit weak growth in the whole plant

**Fig. 1:**
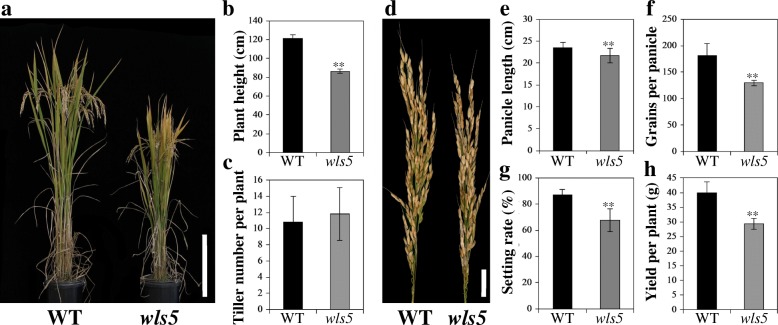
Phenotypic comparison between wild-type (WT) and *wls5* plants. **a** WT (‘93–11’) and *wls5* plants at maturity. Bar = 40 cm. **b**, **c** Statistical analysis of plant height and tiller number between WT and *wls5* plants. Twenty plants were measured. Error bars indicate SD; **P < 0.01 (Student’s *t-*test). **d** Panicle phenotype of WT and *wls5* plants. Bar = 3 cm. **e**-**h** Statistical analysis of panicle length, grains per panicle, setting rate and yield per plant between WT and *wls5* plants. Twenty panicles were measured. Error bars indicate SD; **P < 0.01 (Student’s *t*-test)

The *wls5* mutant was obtained from an ethyl methane sulfonate (EMS) mutant bank of the *indica* rice cultivar ‘93–11’. Under normal growth conditions, *wls5* plants exhibited weak growth (Fig. 1a). Compared with the wild type, tiller number was not altered, but plant height was only about 71.1% that of the wild type at the mature stage (Fig. 1b, c). The *wls5* mutant also had shorter panicles, fewer grains per panicle, and lower seed-setting rate than the wild type (Fig. 1d-g). These reductions in major agronomic traits led to significant yield reduction in *wls5*, to just 73.2% of that in the wild type (*P* < 0.01, Student’s *t-*test; Fig. 1h).

**Fig. 2:**
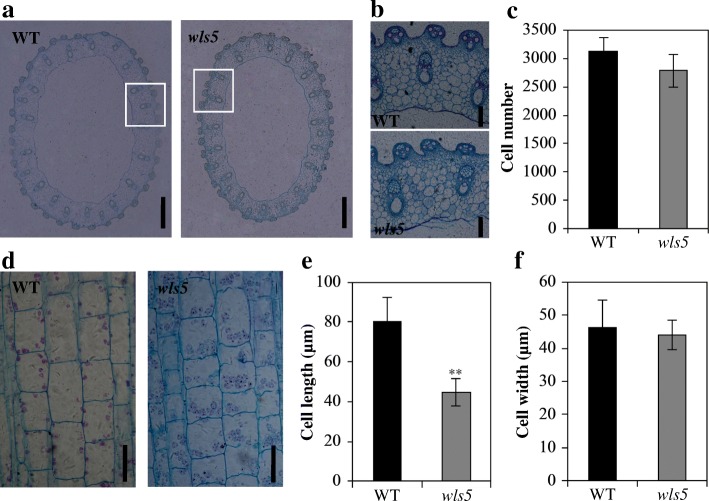
Histological characterization of culms in wild-type (WT) and *wls5* plants. **a** Cross sections of internode II of WT (‘93–11’) and *wls5* plants at heading stage. **b** Magnification of a. Bars = 500 μm (**a**), 50 μm (**b**). **c** Statistical analysis of cell number between WT and *wls5* plants; means ± SD of five independent replicates. **d** Longitudinal sections of internode II of WT and *wls5* plants. Bars = 50 μm. **e**-**f** Statistical analysis of cell length and cell width between WT and *wls5* plants; means ± SD of 30 cells. **P < 0.01 (Student’s *t-*test)

Plant organ size is determined by the number and size of cells, which are regulated by cell division and cell expansion, respectively (Krizek 2009). To investigate the causes of organ size reduction in *wls5* plants, we compared paraffin sections of the second culms of wild-type and *wls5* plants. Cross sections revealed that culm size in *wls5* was smaller than that in the wild type (Fig. 2a, b). Statistical analysis showed that the cell number in *wls5* was only 89.2% of that in the wild type (Fig. 2c). Longitudinal sections of culms revealed a dramatic change in cell size of *wls5* compared with the wild type (Fig. 2d). Cell length in *wls5* was 55.9% of that in the wild type, while cell width was similar in both (Fig. 2e, f). In addition, longitudinal sections of leaves indicated that the development and arrangement of mesophyll cells in *wls5* were also abnormal (Additional file 1: Figure S1).

#### *wls5* undergoes early leaf senescence

**Fig. 3:**
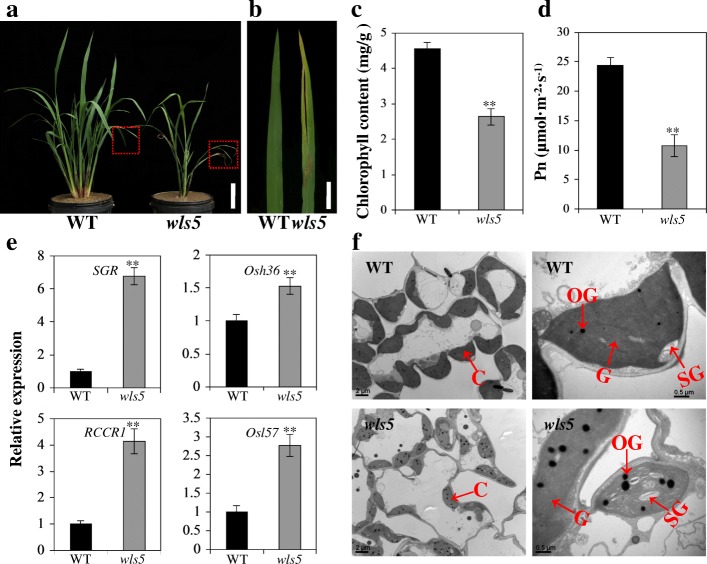
Identification of leaf senescence in *wls5*. **a** Wild-type (WT; ‘93–11’) and *wls5* plants at the tillering stage. Bar = 10 cm. **b** Leaf phenotype of WT and *wls5* plants. Bar = 2 cm. **c** Chlorophyll content of leaves in wild-type and *wls5* plants. Error bars indicate SD, *n* = 10. **P < 0.01 (Student’s *t*-test). **d** Photosynthesis rate of leaves in wild-type and *wls5* plants. Error bars indicate SD, *n* = 15. **P < 0.01 (Student’s *t*-test). **e** Expression of CDGs (*SGR* and *RCCR1*) (left) and other SAGs (*Osh36* and *Osl57*) (right) in WT and *wls5* plants. Error bars indicate SD, *n* = 3. **P < 0.01 (Student’s *t-*test). **f** Transmission electron microscopy of senescing leaves of WT and *wls5* plants at seedling stage. C, chloroplast; OG, osmiophilic granule; SG, starch granule; G, grana thylakoid

In addition to developmental weakness, *wls5* also exhibited an early senescence phenotype, displaying yellow spots at the tip of each leaf at the tillering stage (Fig. 3a, b). The chlorophyll content in *wls5* plants was significantly lower, only 57.9%, of that in the wild type (*P* < 0.01, Student’s *t-*test; Fig. 3c). In addition, the photosynthetic rate (*P*_n_) in *wls5* plants was only 44.3% of that in the wild type (Fig. 3d). To confirm senescence in *wls5* plants, we determined the expression levels of two chlorophyll degradation related genes (CDGs), *STAY-GREEN* (*SGR*) and *RED CHLOROPHYLL CATABOLITE REDUCTASE 1* (*RCCR1*) (Jiang et al. 2007; Tang et al. 2011), and two other senescence-associated genes (SAGs), *Osh36* and *OsI57* (Lee et al. 2001), by reverse-transcription quantitative PCR (RT-qPCR). The *wls5* plants had higher expression levels of these senescence-related genes than wild-type plants (Fig. 3e).

To explore the cause of low chlorophyll levels in *wls5* during senescence, we compared the chloroplast ultrastructure of mesophyll cells in fully expanded leaves of *wls5* and wild-type plants by transmission electron microscopy (TEM). In comparison with the wild type, the number and size of chloroplasts were dramatically lower in leaves of *wls5* plants showing yellow spots (Fig. 3f, left). Furthermore, degenerated thylakoid membrane and a decreased number of grana thylakoid were observed in *wls5* (Fig. 3f, right). The *wls5* mutant cells also contained many more osmiophilic granules than wild-type cells (Fig. 3f, right). These observations suggest that the *wls5* mutant may undergo abnormal chloroplast development.

**Fig. 4:**
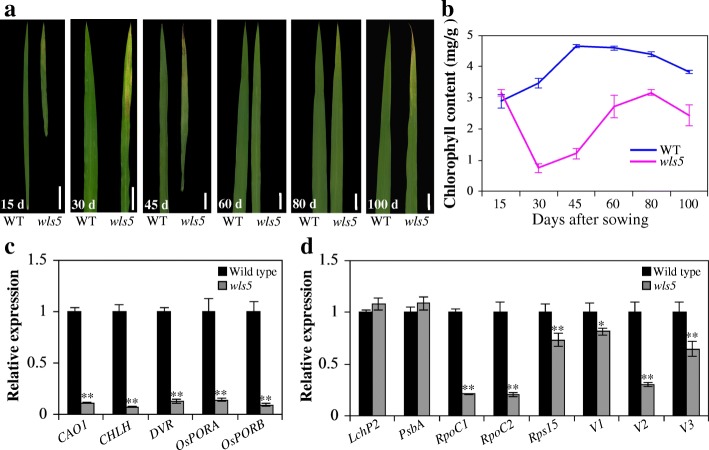
Comparison of leaf senescence over time between wild-type (WT) and *wls5* plants. **a** Leaf phenotype of WT (‘93–11’) and *wls5* plants at different stages (The youngest fully expanded leaf). Bars = 2 cm. **b** Chlorophyll content of leaves in wild-type and *wls5* plants. Error bars indicate SD, *n* = 10. **c**, **d** Expression of chlorophyll synthesis (**c**) and chloroplast development (**d**) related genes in WT and *wls5* plants at tillering stage. Error bars indicate SD, *n* = 3. **P < 0.01 (Student’s *t*-test)

We monitored the leaf senescence phenotype of *wls5* plants throughout plant growth, observing that *wls5* showed early senescence to varying degrees at different growth stages (Fig. 4a). No leaf senescence was visible in *wls5* mutants or wild-type plants during early germination. Around 15 days after germination, the mutant leaf apex exhibited a faint yellow color. With continued development, the yellow spots on *wls5* leaves (30 and 45 days) expanded and became more yellow. When *wls5* plants (60 and 80 days) progressed to the jointing stage, however, the young leaves (100 days) turned green at heading time then exhibited apparent senescence again at the grain-filling stage (Fig. 4a). Consistent with the leaf senescence phenotype, the chlorophyll content of *wls5* plants decreased dramatically at the tillering stage, then increased until heading stage, and subsequently decreased rapidly again at maturation (Fig. 4b). These results indicate that internal or external factors influenced the leaf senescence process in *wls5* mutants in different growth stages.

Leaf senescence is usually accompanied by a change in expression of numerous genes, including those for chlorophyll synthesis and chloroplast development (Wang et al. 2015a, b). Therefore, we detected the expression levels of chlorophyll synthesis related genes (*CAO1*, *CHLH*, *DVR*, *OsPORA*, and *OsPORB*) and chloroplast development related genes (*LchP2*, *PsbA*, *RpoC1*, *RproC2*, *Rps15*, *V1*, *V2*, and *V3*) in *wls5* and wild-type plants. Expression levels of *CAO1*, *CHLH*, *DVR*, *OsPORA*, *OsPORB*, *RpoC1*, *RproC2*, *Rps15*, *V1*, *V2*, and *V3* were significantly lower in *wls5* plants compared to those in the wild type (*P* < 0.01, Student’s *t*-test; Fig. 4c, d), further supporting the notion that early leaf senescence and abnormal chloroplast development occurred in *wls5* plants.

#### ROS accumulation is enhanced in *wls5* plants

**Fig. 5:**
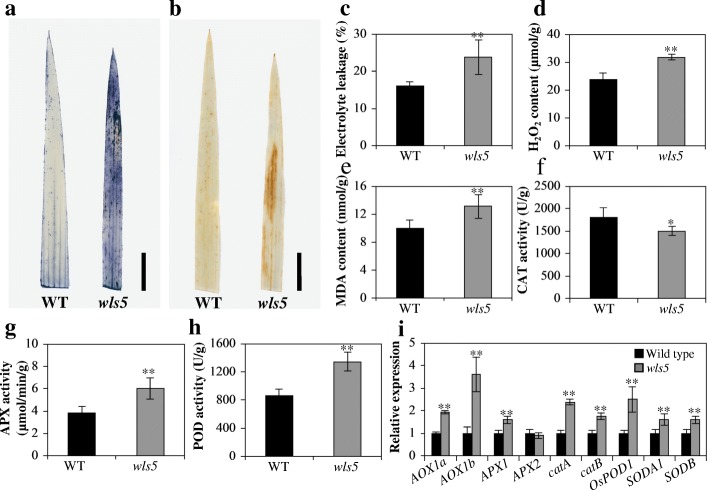
ROS accumulation in wild-type (WT) and *wls5* plants. **a**, **b** NBT (**a**) and DAB (**b**) staining of leaves from WT (‘93–11’) and *wls5* plants at seedling stage. Bars = 1 cm. **c**-**h** Statistical analysis of electrolyte leakage (**c**), H_2_O_2_ content (**d**), MDA content (**e**), CAT activity (**f**), APX activity (**g**) and POD activity (**h**) in leaves of WT and *wls5* plants. Mean ± SD of five independent replicates, **P < 0.01, *P < 0.05 (Student’s *t-*test). i Relative expression levels of ROS detoxification related genes in WT and *wls5* plants; mean ± SD of three independent replicates. **P < 0.01 (Student’s *t-*test)

ROS accumulation promotes premature senescence (Roy et al. 2012). We therefore used the same part of the top leaves from *wls5* and wild-type plants at the seedling stage to perform nitro blue tetrazolium (NBT) staining and 3,3′-diaminobenzidine (DAB) staining tests. As shown in Fig. 5a, extensive NBT staining was observed in *wls5* plants, whereas staining was minimal in wild-type leaves. Similarly, there was little sign of DAB staining in wild-type leaves, but a brown color was visible in *wls5* mutant leaves, consistent with the area of leaf senescence (Fig. 5b). These results indicated that ROS accumulated in *wls5* plants. We then measured electrolyte leakage, an indicator of plant cell membrane damage (Blum and Ebercon 1981), and concentrations of senescence-related substances, including the ROS hydrogen peroxide (H_2_O_2_) and the lipid oxidation byproduct malondialdehyde (MDA). Electrolyte leakage in *wls5* plants was 48.6% higher than that in the wild type (Fig. 5c). Similarly, higher concentrations of H_2_O_2_ and MDA were observed in *wls5* leaves than in wild-type leaves (Fig. 5d, e), indicating that ROS accumulation did occur during premature leaf senescence in *wls5* mutants.

ROS scavenging enzymes, such as catalase (CAT), ascorbate peroxidase (APX), superoxide dismutase (SOD) and peroxidase (POD), play significant regulatory roles in plant senescence (Miller et al. 2010). We therefore quantified the activity of these enzymes. In comparison with the wild type, CAT activity was significantly lower in *wls5* (*P* < 0.05, Student’s *t-*test; Fig. 5f). By contrast, the activities of APX and POD were much higher in *wls5* leaves (6.0 μmol/min/g and 1344 U/mg fresh weight, respectively) than in wild-type leaves (3.8 μmol/min/g and 864 U/mg fresh weight, respectively) (Fig. 5g, h). The *wls5* and wild-type plants showed no significant difference in SOD activity (318.6 and 316.6 U/mg fresh weight, respectively). As ROS-scavenging systems have an important role in ROS detoxification (Tan et al. 2014), we detected expression levels of ROS scavenging related genes. Expression levels of *AOX1a*, *AOX1b*, *APX1*, *catA*, *catB*, *OsPOD1*, *SODA*, and *SODB* were significantly higher in *wls5* plants than in the wild type (*P* < 0.01, Student’s *t-*test; Fig. 5i).

#### *wls5* affects the density of leaf stomata

**Fig. 6:**
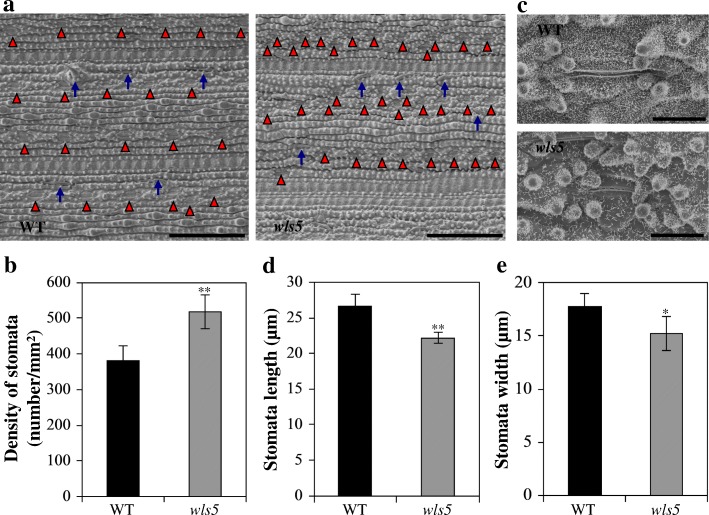
Observation of stomata in wild-type (WT) and *wls5* plants. **a** Stomatal density of WT (‘93–11’) and *wls5* leaves at tillering stage. Red triangles and blue arrows indicate the positions of stomata and setae, respectively. Bars = 100 μm. **b** Statistical analysis of stomatal density. **c** Morphological characteristics of stomata of WT and *wls5* plants at tillering stage. Bars = 10 μm. **d**, **e** Statistical analysis of stomata length (**d**) and stomata width (**e**). Three independent replicates for stomatal density, and 20 independent replicates for stomata size. **P < 0.01 (Student’s *t*-test)

The rice early senescence mutant *early senescence 1* has greater stomatal density than wild-type rice (Rao et al. 2015). To examine whether *wls5* has the same phenotype, we compared leaves of *wls5* and wild-type plants by scanning electron microscopy. Stomatal density was 36.4% higher in *wls5* plants (518.3/mm^2^) than in wild-type leaves (380.0/mm^2^) (Fig. 6a, b). Stomatal length and width were 22.2 and 15.2 μm, respectively, in *wls5* plants, much smaller than the 26.6 and 17.8 μm observed in the wild type (Fig. 6c-e). These results indicate that *wls5* affects the density of leaf stomata in rice. In addition, the tips of trichomes and the setae were severely degraded on the leaf surface of *wls5* mutants (Fig. 6a, c).

#### Hormone levels are disturbed in *wls5* mutants

**Fig. 7:**
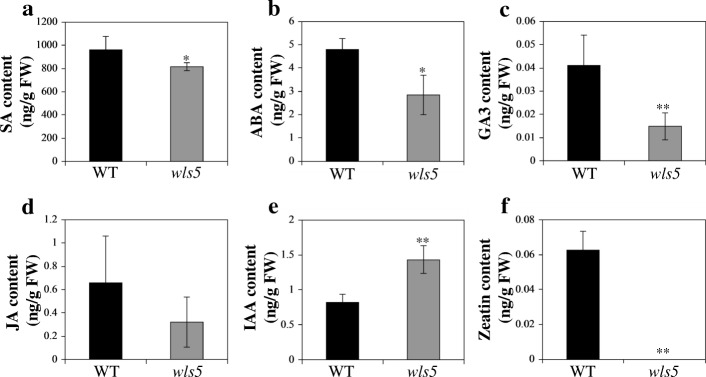
Comparison of endogenous hormone contents between wild-type (WT) and *wls5* plants. SA (**a**), ABA (**b**), GA3 (**c**), JA (**d**), IAA (**e**) and zeatin (**f**) content in leaves of WT (‘93–11’) and *wls5* plants at tillering stage. Means ± SD of three independent replicates. *P < 0.05; **P < 0.01 (Student’s *t*-test)

Phytohormone levels regulate leaf senescence (Jan et al. 2019). For example, gibberellic acid (GA) inhibits senescence (Yu et al. 2009), and auxins decrease the expression of *SAG12*, thereby delaying senescence (Mueller-Roeber and Balazadeh 2014). We therefore measured endogenous hormone contents in leaves at the tillering stage. Compared with wild type, the salicylic acid (SA; *P* < 0.05), abscisic acid (ABA; *P* < 0.05) and GA (*P* < 0.01) contents of *wls5* mutants were significantly lower, being only about 84.9%, 59.1% and 35.9% of those in wild-type plants (Student’s *t*-test; Fig. 7a-c). The jasmonic acid (JA) content in *wls5* was also lower than that in the wild type (Fig. 7d). By contrast, *wls5* plants had a higher indole-3-acetic acid (IAA) content than wild-type plants: 1.43 and 0.82 ng/g fresh weight, respectively (Fig. 7e). The zeatin content was 0.06 ng/g fresh weight in the wild type, but zeatin levels were too low to measure in *wls5* mutants (Fig. 7f). These results indicate that endogenous hormone levels were disturbed in *wls5* mutants.

#### RNA sequencing analysis of *wls5* mutants

**Fig. 8:**
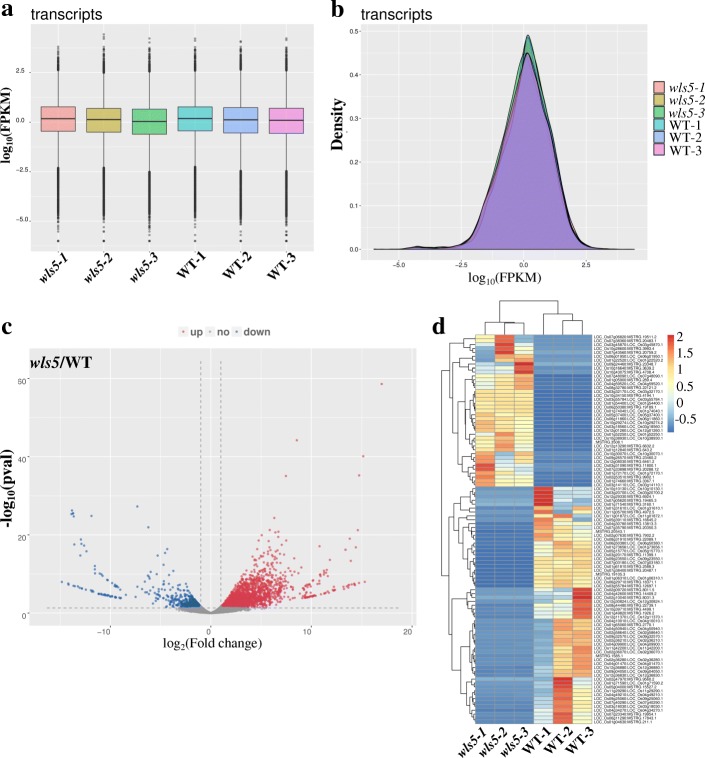
RNA-seq analysis of wild-type (WT) and *wls5* plants. mRNA was purified from total RNA isolated from tillering-stage plants of WT (‘93–11’) and *wls5*. **a** Transcript expression boxplots of WT and *wls5* plants. **b** Transcript expression density of WT and *wls5*. **c** Volcano plot showing the overall alterations in gene expression between WT and *wls5*. **d** Cluster analysis of differentially expressed genes in WT and *wls5*. Red represents highly expressed genes. Blue represents poorly expressed genes

Regulation of leaf senescence is highly complex, with many specific genes involved (Lin et al. 2015). We performed RNA sequencing (RNA-seq) to analyze the effect of the *wls5* mutation on gene expression. Leaf tip samples of *wls5* and wild-type plants at tillering stage were selected for sequencing analysis with three replicates. Boxplots and density profiles of transcript levels in wild-type and *wls5* plants indicated good reproducibility among biological replicates (Fig. 8a, b). More than 50 million reliable, clean reads were obtained from *wls5* and wild-type samples, representing 2606 up-regulated genes and 840 repressed genes in *wls5* compared to the wild type (Fig. 8c, d)*.* Gene ontology and Kyoto Encyclopedia of Genes and Genomes pathway enrichment analyses indicated that genes involved in oxidoreductase activity, the oxidation-reduction process, response to ABA, multicellular organism development and ABA-activated signaling pathways were altered in *wls5* plants (Additional file 1: Figure S2-S4).

#### Candidate gene analysis of *WLS5*

We employed a map-based cloning approach to isolate the gene responsible for the *wls5* phenotype. A mapping population was generated by crossing *wls5* with the *japonica* cultivar ‘Nipponbare’. All F_1_ plants showed a normal phenotype matching that of the wild type. A segregating population of 1088 F_2_ plants was obtained, among which 821 plants displayed the normal wild-type phenotype and 267 plants exhibited the early leaf senescence phenotype similar to *wls5*, fitting a typical segregation ratio of 3:1 (χ^2^ = 0.12 < χ^2^_0.05_ = 3.84). This suggested that *wls5* was controlled by a single recessive nuclear gene.

**Fig. 9:**
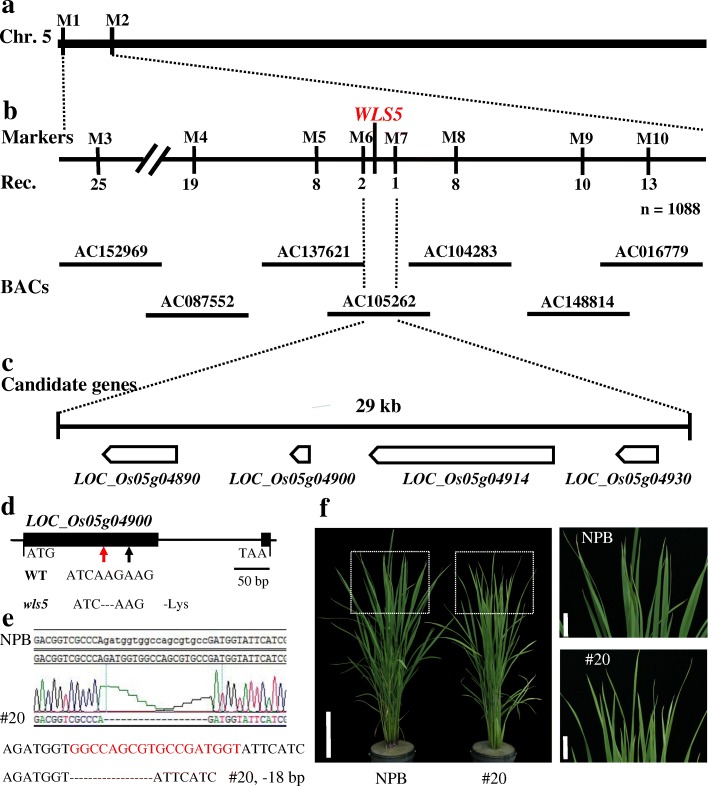
Candidate gene analysis for *WLS5*. **a** Location of *WLS5* on rice chromosome 5. **b** Coarse linkage map of *WLS5*. Markers used for mapping are indicated. Numbers below lines indicate number of recombinants. **c** Candidate genes for *WLS5*. **d** *LOC_Os05g04900* structure and sequence variations between wild type and *wls5*. Black and red arrows indicate mutation site and target knockout position, respectively. **e**, **f** *LOC_Os05g04900* knockout in ‘Nipponbare’ (NPB). CRISPR/Cas9-induced 18-bp deletion in *LOC_Os05g04900* (**e**). Phenotype of NPB and line #20. Bars = 25 cm (left) and 12.5 cm (right) (**f**)

Twenty-one F_2_ plants with the *wls5* phenotype were used for primary mapping, which located *WLS5* on the short arm of chromosome 5 between markers M1 and M2 (Fig. 9a). To further narrow the *WLS5* region, we developed a number of additional insertion/deletion (InDel) markers between M1 and M2, as shown in Additional file 2: Table S1. These allowed mapping of *WLS5* to a region of 29 kb between markers M6 and M7, with two and one recombinants, respectively (Fig. 9b). This region does not contain any known senescence-related loci. Four genes were predicted within this 29-kb region, based on the Rice Genome Annotation Project (http://rice.plantbiology.msu.edu/) (Fig. 9c), *LOC_Os05g04890*, *LOC_Os05g04900*, *LOC_Os05g04914*, and *LOC_Os05g04930*. Sequence alignment of these four genes from wild-type and *wls5* plants identified only a 3-bp deletion in the coding sequence of *LOC_Os05g04900* in *wls5*, which led to a lysine (Lys) deletion in the protein (Fig. 9d). *LOC_Os05g04900* encodes a predicted protein of 87 amino acids with no predicted function; however, the protein has two potential transmembrane domains.

To test whether *LOC_Os05g04900* is responsible for the *wls5* phenotype, we used genome editing to generate an additional mutant allele. Using CRISPR/Cas9, we obtained a homozygous mutant, line #20, with an 18-bp deletion in *LOC_Os05g04900*, in the ‘Nipponbare’ genetic background (Fig. 9e). This genetically edited line showed similar leaf senescence phenotype to the *wls5* mutant (Fig. 9f), indicating that *LOC_Os05g04900* might be the candidate gene for *WLS5*.

## Discussion

As the final phase of plant development, senescence is under the control of a highly regulated genetic program (Lim et al. 2007). A growing number of leaf senescence-associated genes have been cloned and functionally characterized in rice (Mitchell and Sheehy 2006; Yamada et al. 2014; Wu et al. 2016; Yang et al. 2016a; Yang et al. 2016b; Zhao et al. 2016; Deng et al. 2017; Leng et al. 2017b; Mao et al. 2017; Hong et al. 2018). However, the molecular mechanism underlying leaf senescence remains poorly understood. In this study, we characterized a novel premature senescence mutant, *wls5*, which also showed weak plant growth.

Previous studies have identified and characterized several mutants involved in both premature leaf senescence and weak plant growth in rice (Wu et al. 2016; Xie et al. 2016; El Mannai et al. 2017; Leng et al. 2017b; He et al. 2018; Hong et al. 2018). In Arabidopsis, mutations in some genes also lead to similar phenotype. For instance, the *mod1* (*mosaic death1*) mutant displayed multiple morphological phenotypes, including chlorotic and semidwarfism (Mou et al. 2000). The T-DNA knockout plants of *RPI2* gene (*At2g01290*) were smaller than its wild-type and exhibited chlorosis phenotype (Xiong et al. 2009), and *PIC1* (*permease in chloroplasts 1*) mutants showed severely impaired plastid development and plant growth (Duy et al. 2011). These results suggest that leaf senescence and plant growth processes might be regulated by the same genetic factors in plants. Here, *wls5* mutant plants not only showed leaf senescence throughout their growth (Fig. 3a, b, Fig. 4a), but also exhibited dwarfism and small organs (Fig. 1, Fig. 3a). Similar to previously characterized mutants, leaf senescence in *wls5* was directly related to low chlorophyll levels and high ROS accumulation (Fig. 3c, Fig. 4b, Fig. 5), and abnormal growth of *wls5* was caused by a reduction in cell number and small cell size compared to the wild type (Fig. 2). Thus, one genetic factor affects these two processes, although the underlying mechanism remains largely unclear and requires further study. The *wls5* mutant provides ideal material for this research.

Leaf senescence is generally accompanied by a decrease in chlorophyll levels and an increase in various ROS (He et al. 2018; Hong et al. 2018). In the present study, chlorophyll was degraded in *wls5* (Fig. 3c, Fig. 4b), and chloroplast structure was also abnormal (Fig. 3f). Meanwhile, expression levels of genes involved in chlorophyll synthesis and chloroplast development were significantly down-regulated in *wls5* plants compared to those in the wild type (Fig. 4c, d). Therefore, we speculate that degradation of chlorophyll and chloroplasts plays an important role in leaf senescence in *wls5* plants. Various metabolic processes generate ROS in plants, causing oxidative damage to thylakoid membranes and other cellular components (Apel and Hirt 2004); (Wang et al. 2015a, b). Here, we found greater accumulation of superoxide anions (O_2_^−^) and H_2_O_2_ in *wls5* plants than wild-type plants (Fig. 5a, b, d). Consistent with this, more electrolyte leakage was observed in *wls5* than in the wild type (Fig. 5c). In addition, APX and POD activities were higher in *wls5* than in wild-type plants (Fig. 5g, h). RNA-seq indicated that expression of genes involved in oxidoreductase activity and oxidation-reduction processes was remarkably altered in *wls5* plants (Additional file 1: Figure S2-S4). Taking these finding together, it is clear that leaf senescence in *wls5* plants involves ROS.

Phytohormones play important regulatory roles in promoting or delaying leaf senescence (Jan et al. 2019). In our study, *wls5* mutants had lower SA, GA3, ABA, JA, and zeatin contents, and higher IAA content compared to the wild type (Fig. 7). These altered levels of endogenous hormones in *wls5* mutants may contribute to its leaf senescence phenotype. Furthermore, genes responsive to ABA and involved in ABA-activated signaling pathways were dramatically up-regulated or down-regulated in *wls5* plants compared to the wild type (Additional file 1: Figure S4). However, ABA is an important endogenous factor that induces leaf senescence in plants (Lin et al. 2015). This is not consistent ith the low ABA content in *wls5* plants, indicating that leaf senescence in the *wls5* mutant may not involve ABA signaling pathways.

Using a map-based cloning approach, we fine mapped *WLS5* to a 29-kb region containing four predicted genes on the short arm of chromosome 5 (Fig. 9a-c). No evidence currently suggests that these four genes are associated with premature leaf senescence or weakness in plant growth; thus, *wls5* is most likely a novel mutant for weakness and leaf senescence in rice. Sequence comparison of these four genes between the wild type and *wls5* identified only a 3-bp deletion in the coding sequence region of *LOC_Os05g04900*, a gene encoding an expressed protein of unknown function, in *wls5* (Fig. 9d). This mutation caused a lysine (Lys) deletion in the predicted protein. Given the leaf senescence phenotype of the *LOC_Os05g04900* knockout line (Fig. 9e, f), we inferred that *LOC_Os05g04900* is the candidate gene for *WLS5*. By multiple sequences alignment and phylogeny evolution analysis of *LOC_Os05g04900* sequence with the other species, we found no homologous genes to *WLS5*/*wls5* except for the *GRMZM2G111850* in maize, whose gene function is also unidentified. Therefore, *WLS5*/*wls5* is a new genetic factor for growth and senescence in plants, but its molecular function needs further study.

## Conclusions

A novel mutant involved in plant development and leaf senescence was identified in rice. *LOC_Os05g04900*, encoding a protein of unknown function, is the candidate gene for *WLS5*. Further molecular study of *wls5* will uncover the functional roles of this gene in plant growth and leaf senescence.

## Methods

### Plant materials and growth conditions

The rice (*Oryza sativa*) *wls5* mutant was isolated from the ethyl methane sulfonate treated *indica* cultivar ‘93–11’. An F_2_ mapping population was generated from a cross between *wls5* and the *japonica* cultivar ‘Nipponbare’. All plants were grown in paddy fields in either Zhejiang or Hainan provinces in China (2015–2018).

### Paraffin sectioning and microscopic analysis

Internodes and leaves of wild-type and *wls5* plants were collected at heading stage and fixed in 50% ethanol, 0.9 M glacial acetic acid, and 3.7% formaldehyde overnight at 4 °C. These samples were then dehydrated with a graded series of ethanol, infiltrated with xylene and embedded in paraffin (Sigma). The specimens were sectioned (8 mm thickness) with a Leica RM2245 microtome, then transferred onto poly-L-Lys-coated glass slides, deparaffinized in xylene and dehydrated through an ethanol series. After staining with 1% safranin (Amresco) and 1% Fast Green (Amresco), sections were dehydrated through an ethanol series, infiltrated with xylene and finally mounted beneath a coverslip (Ren et al. 2016). Light microscopy was performed using a Nikon SMZ1500 microscope.

### Measurement of pigment content and photosynthetic rate

Fresh leaf samples (0.05 g) of wild-type and *wls5* plants were cut into small segments and incubated with 5 ml 80% acetone in the dark for 24 h. Absorbance of the supernatant was then measured with an ultraviolet spectrophotometer (DU800, BECKMAN, Fullerton, USA) at 470, 645, and 663 nm (Chen et al. 2018; Wang et al. 2018). Total chlorophyll was determined according to the methods of Arnon (1949) and Wellburn (1994). Ten biological replicates were analyzed for each condition.

Photosynthetic rates of wild-type and *wls5* plants grown in Zhejiang were measured at 65 days post-sowing at 10:30 am on a sunny day using a LI-6400 portable photosynthesis device (LICOR, USA). Fifteen biological replicates were included.

### TEM analysis

TEM assays were performed according to the methods described by Leng et al. (2017a). Fresh leaves of wild-type and *wls5* plants were fixed in 2.5% glutaraldehyde in phosphate-buffered saline (PBS, 137 mM NaCl, 2.7 mM KCl, 8 mM Na_2_HPO_4_, and 2 mM KH_2_PO_4_, pH 7.4) for at least 4 h and washed in PBS three times. The samples were then postfixed with 1% (*w*/*v*) OsO_4_ for 2 h after extensive washing in PBS, dehydrated with a graded ethanol series and infiltrated with Spurr Kit (Sigma). The specimens were sectioned (70 nm ultrathin) with a Leica EM UC7 ultramicrotome, and sections were stained with uranyl acetate and alkaline lead citrate for 10 min. TEM was performed using a Hitachi model H-7650.

### Histochemical staining and ROS-scavenging enzyme assays

DAB and NBT staining were performed on leaves from wild-type and *wls5* plants to detect H_2_O_2_ and superoxide anions, as described by Blum and Ebercon (1981). Electrolyte leakage was determined in accordance with a previous study (Zhou and Guo, 2009). H_2_O_2_, MDA content and CAT, APX, and POD activity of wild-type and *wls5* mutant leaves were measured using an Assay Kit (Suzhou Keming Biotechnology Co., Ltd.).

### RT-qPCR analysis

Total RNA was extracted from fresh leaf samples of wild-type and *wls5* plants using a Micro RNA Extraction kit (Axygen) and reverse transcribed into cDNA using a Rever Tra Ace qPCR-RT kit (TOYOBA, Japan). qPCR was conducted on an ABI PRISM 7900HT Sequence Detector (Applied Biosystems) according to the manufacturer’s instructions. Primers for RT-qPCR are listed in Additional file 2: Table S1. The rice *UBQ5* gene was used as an internal control. Three biological replicates of each sample were prepared.

### Hormone extraction and determination of hormone levels

Levels of SA, ABA, GA3, JA, IAA, and zeatin were determined according to a previously described method with little modification (You et al. 2016). Fresh leaf samples (1.0 g) of wild-type and *wls5* plants were ground in pre-cooled extraction buffer (10 ml) and shaken at 4 °C for 30 min. Dichloromethane (20 mL) was added, and the sample was shaken at 4 °C for 30 min and then centrifuged at 16,020 *g* for 5 min. The organic phase was extracted and dried under liquid nitrogen. Pellets were dissolved in 150 mL methanol (0.1% methane acid) and filtered with a 0.22-mm filter membrane. The purified product was then subjected to high-performance liquid chromatography tandem mass spectrometry (HPLC-MS/MS) analysis. The injection volume was 2 mL. Mass spectrometry conditions were as follows: spray voltage was 4500 V; pressure of the air curtain, nebulizer and aux gas was 15, 65 and 70 psi, respectively; and atomizing temperature was 400 °C.

### RNA-seq analysis

Total RNA was extracted using a TRK1001 Total RNA Purification Kit (LC Science, Houston, TX, USA) following the manufacturer’s procedure, then quantified using a Bioanalyzer 2100 and RNA 6000 Nano LabChip Kit (Agilent, CA, USA) with RIN number > 7.0. Poly(A) mRNA was isolated from approximately 5 μg total RNA by using magnetic beads with poly-T oligo attached (Invitrogen). cDNA was synthesized using random hexamer primers. A library was constructed and sequenced using an Illumina Hiseq 2000/2500 (LC Sciences, USA). More than 50 million reads from wild-type and *wls5* plants were obtained. Gene ontology analysis was performed using GOseq (Young et al. 2010). Pathway enrichment analysis was performed using the Kyoto Encyclopedia of Genes and Genomes database (Kanehisa et al. 2008).

### Map-based cloning

A fine-mapping population was generated from a cross between a *wls5* mutant (in the *indica* background) and the *japonica* rice cultivar ‘Nipponbare’. A total of 267 individual plants from a segregating population of 1088 F_2_ plants showed the same phenotype as the *wls5* mutant, and were sampled for mapping. The initial location of *wls5* was determined using 163 simple sequence repeat (SSR) markers scattered across the 12 rice chromosomes (http://www.gramene.org). For fine mapping, eight InDel markers between the M1 and M2 markers were designed based on genomic DNA sequences from ‘Nipponbare’ and the *indica* rice variety ‘93–11’, to narrow down the *wls5* region. All markers used are detailed in Additional file 2: Table S1.

### CRISPR/Cas9 vector construction and rice transformation

CRISPR/Cas9 genome editing vector construction was performed as described by (Wang et al. 2015a, b). One specific target guide RNA sequence, GATGGTGGCCAGCGTGCCGATGG, located in the first exon was selected to generate mutants of *LOC_Os05g04900*. Primers for vector construction are listed in Additional file 2: Table S1. The fragment was inserted into a pC1300-UBI:Cas9 vector and introduced into ‘Nipponbare’ by *Agrobacterium tumefaciens* (EHA105) mediated transformation. Twenty independent transgenic plants (T_0_) were obtained and sequenced. One homozygous mutant, line #20, and its wild type (‘Nipponbare’) were grown in the field for phenotype determination.

## Additional files

Additional file 1: **Figure S1.** Histological characterization of leaves in wild-type and *wls5* plants. **a**, **b** Cross sections of leaves of wild-type (‘93-11’) and *wls5*. **c**-**f** Longitudinal sections of leaves of wild-type and *wls5* plants. Scale bar = 50 μm. **Figure S2.** Statistics of GO enrichment analysis of differential expression genes between wild-type (WT) and *wls5* plants. mRNA was purified from total RNA isolated from tillering-stage plants of WT (‘93-11’) and *wls5*. **Figure S3.** Statistics of pathway enrichment analysis of differential expression genes between wild-type (WT) and *wls5* plants. mRNA was purified from total RNA isolated from tillering-stage plants of WT (‘93-11’) and *wls5.* **Figure S4.** The number of differential expression genes involved in different biological processes, cellular components and molecular functions. mRNA was purified from total RNA isolated from tillering-stage plants of WT (‘93-11’) and *wls5. (PDF 2035 kb)*

Additional file 2: **Table S1.** The list of primers used in this study (PDF 77 kb)

## Abbreviations

CRISPR/Cas9: Clustered regularly interspaced short palindromic repeats and CRISPR-associated protein 9
DAB: 3,3′-diaminobenzidine
NBT: Nitro blue tetrazolium
qPCR: Quantitative PCR
TEM: Transmission electron microscopy

## References

[CR1] Apel K, Hirt H (2004). Reactive oxygen species: metabolism, oxidative stress, and signal transduction. Annu Rev Plant Biol.

[CR2] Arnon DI (1949). Copper enzymes in isolated chloroplasts. Polyphenoloxidase in Beta vulgaris. Plant Physiol.

[CR3] Blum A, Ebercon A (1981). Cell membrane stability as a measure of drought and heat tolerance in wheat 1. Crop Sci.

[CR4] Chen P, Hu HT, Zhang Y, Wang ZW, Dong GJ, Cui YT, Qian Q, Ren DY, Guo LB (2018). Genetic analysis and fine-mapping of a new rice mutant, *white and lesion mimic leaf1*. Plant Growth Regul.

[CR5] Deng L, Qin P, Liu Z, Wang G, Chen W, Tong J, Xiao L, Tu B, Sun Y, Yan W, He H, Tan J, Chen X, Wang Y, Li S, Ma B (2017). Characterization and fine-mapping of anovel premature leaf senescence mutant *yellow leaf and dwarf 1* in rice. Plant Physiol Biochem.

[CR6] Duan Y, Li S, Chen Z, Zheng L, Diao Z, Zhou Y, Lan T, Guan H, Pan R, Xue Y, Wu W (2012). Dwarf and deformed flower 1, encoding an F-box protein, is critical forvegetative and floral development in rice (Oryza sativa L.). Plant J.

[CR7] Duy D, Stübe R, Wanner G, Philippar K (2011). The chloroplast permease PIC1 regulates plant growth and development by directing homeostasis and transport of iron. Plant Physiol.

[CR8] El MY, Akabane K, Hiratsu K, Satoh-Nagasawa N, Wabiko H (2017) The NAC transcription factor gene OsY37 (ONAC011) promotes leaf senescence and accelerates heading time in rice. Int J Mol Sci 18(10) 10.3390/ijms18102165 PMC5666846 29039754

[CR9] He Yan, Zhang Zhihong, Li Liangjian, Tang Shaoqing, Wu Jian-Li (2018). Genetic and Physio-Biochemical Characterization of a Novel Premature Senescence Leaf Mutant in Rice (Oryza sativa L.). International Journal of Molecular Sciences.

[CR10] Hong Y, Zhang Y, Sinumporn S, Yu N, Zhan X, Shen X, Chen D, Yu P, Wu W, Liu Q, Cao Z, Zhao C, Cheng S, Cao L (2018). Premature leaf senescence 3, encoding a methyltransferase, is required for melatonin biosynthesis in rice. Plant J.

[CR11] Jan S, Abbas N, Ashraf M, Ahmad P (2019). Roles of potential plant hormones and transcription factors in controlling leaf senescence and drought tolerance. Protoplasma.

[CR12] Jiang H, Li M, Liang N, Yan H, Wei Y, Xu X, Liu J, Xu Z, Chen F, Wu G (2007). Molecular cloning and function analysis of the stay green gene in rice. Plant J.

[CR13] Kanehisa M, Araki M, Goto S, Hattori M, Hirakawa M, Itoh M, Katayama T, Kawashima S, Okuda S, Tokimatsu T, Yamanishi Y (2008). KEGG for linking genomes to life and the environment. Nucleic Acids Res.

[CR14] Ke S, Liu S, Luan X, Xie XM, Hsieh TF, Zhang XQ (2019). Mutation in a putative glycosyltransferase-like gene causes programmed cell death and early leaf senescence in rice. Rice.

[CR15] Krizek BA (2009). Making bigger plants: key regulators of final organ size. Curr Opin Plant Biol.

[CR16] Lee RH, Wang CH, Huang LT, Chen SC (2001). Leaf senescence in rice plants: cloning and characterization of senescence up-regulated genes. J Exp Bot.

[CR17] Leng Y, Yang Y, Ren D, Huang L, Dai L, Wang Y, Chen L, Tu Z, Gao Y, Li X, Zhu L, Hu J, Zhang G, Gao Z, Guo L, Kong Z, Lin Y, Qian Q, Zeng D (2017). A rice *PECTATELYASE-LIKE* gene is required for plant growth and leaf senescence. Plant Physiol.

[CR18] Leng Yujia, Ye Guoyou, Zeng Dali (2017). Genetic Dissection of Leaf Senescence in Rice. International Journal of Molecular Sciences.

[CR19] Lim PO, Kim HJ, Nam HG (2007). Leaf senescence. Annu Rev Plant Bio.

[CR20] Lin M, Pang C, Fan S, Song M, Wei H, Yu S (2015). Global analysis of the *Gossypium hirsutum* L. transcriptome during leaf senescence by RNA-Seq. BMC Plant Biol.

[CR21] Liu X, Wei X, Sheng Z, Jiao G, Tang S, Luo J, Hu P (2016). Polycomb protein OsFIE2 affects plant height and grain yield in rice. PLoS One.

[CR22] Mao C, Lu S, Lv B, Zhang B, Shen J, He J, Luo L, Xi D, Chen X, Ming F (2017). A rice NAC transcription factor promotes leaf senescence via ABA biosynthesis. Plant Physiol.

[CR23] Miller G, Suzuki N, Ciftci-Yilmaz S, Mittler R (2010). Reactive oxygen species homeostasis and signalling during drought and salinity stresses. Plant Cell Environ.

[CR24] Mitchell PL, Sheehy JE (2006). Supercharging rice photosynthesis to increase yield. New Phytol.

[CR25] Mou Z, He Y, Dai Y, Liu X, Li J (2000). Deficiency in fatty acid synthase leads to premature cell death and dramatic alterations in plant morphology. Plant Cell.

[CR26] Mueller-Roeber B, Balazadeh S (2014). Auxin and its role in plant senescence. J Plant Growth Regul.

[CR27] Pan S, Rasul F, Li W, Tian H, Mo Z, Duan M, Tang X (2013). Roles of plant growth regulators on yield, grain qualities and antioxidant enzyme activities in super hybrid rice (Oryza sativa L.). Rice.

[CR28] Rao Y, Yang Y, Xu J, Li X, Leng Y, Dai L, Huang L, Shao G, Ren D, Hu J, Guo L, Pan J, Zeng D (2015). *EARLY SENESCENCE1* encodes a SCAR-LIKE PROTEIN2 that affects water loss in rice. Plant Physiol.

[CR29] Ren D, Rao Y, Huang L, Leng Y, Hu J, Lu M, Zhang G, Zhu L, Gao Z, Dong G, Guo L, Qian Q, Zeng D (2016). Fine mapping identifies a new QTL for brown rice rate in rice (*Oryza sativa* L.). Rice.

[CR30] Roy N, Bagchi S, Raychaudhuri P (2012). Damaged DNA binding protein 2 in reactive oxygen species (ROS) regulation and premature senescence. Int J Mol Sci.

[CR31] Sakamoto T, Matsuoka M (2008). Identifying and exploiting grain yield genes in rice. Curr Opin Plant Biol.

[CR32] Tan J, Tan Z, Wu F, Sheng P, Heng Y, Wang X, Ren Y, Wang J, Guo X, Zhang X, Cheng Z, Jiang L, Liu X, Wang H, Wan J (2014). A novel chloroplast-localized pentatricopeptide repeat protein involved in splicing affects chloroplast development and abiotic stress response in rice. Mol Plant.

[CR33] Tang Y, Li M, Chen Y, Wu P, Wu G, Jiang H (2011). Knockdown of *OsPAO* and *OsRCCR1* cause different plant death phenotypes in rice. J Plant Physiol.

[CR34] Wang C, Shen L, Fu Y, Yan C, Wang K (2015). A simple CRISPR/Cas9 system for multiplex genome editing in rice. J Genet Genomics.

[CR35] Wang Z, Wang Y, Hong X, Hu D, Liu C, Yang J, Li Y, Huang Y, Feng Y, Gong H, Li Y, Fang G, Tang H, Li Y (2015). Functional inactivation of UDP-*N-acetylglucosamine* pyrophosphorylase 1 (UAP1) induces early leaf senescence and defence responses in rice. J Exp Bot.

[CR36] Wang ZW, Lv J, Xie SZ, Zhang Y, Qiu ZN, Chen P, Cui YT, Niu YF, Hu SK, Jiang HZ, Sheng-zhen Ge SZ, Trinh HP, Lei KR, Bai WQ, Zhang Y, Guo LB, Ren DY (2018). OsSLA4 encodes a pentatricopeptide repeat protein essential for early chloroplast development and seedling growth in rice. Plant Growth Regul.

[CR37] Wellburn AR (1994). The spectral determination of chlorophylls a and b, as well as total carotenoids, using various solvents with spectrophotometers of different resolution. J Plant Physiol.

[CR38] Wu L, Ren D, Hu S, Li G, Dong G, Jiang L, Hu X, Ye W, Cui Y, Zhu L, Hu J, Zhang G, Gao Z, Zeng D, Qian Q, Guo L (2016). Down-regulation of a nicotinate phosphoribosyltransferase gene, *OsNaPRT1*, leads to withered leaf tips. Plant Physiol.

[CR39] Xie Q, Liang Y, Zhang J, Zheng H, Dong G, Qian Q, Zuo J (2016). Involvement of a putative bipartite transit peptide in targeting rice pheophorbide a oxygenase into chloroplasts for chlorophyll degradation during leaf senescence. J Genet Genomics.

[CR40] Xiong Y, DeFraia C, Williams D, Zhang X, Mou Z (2009). Deficiency in a cytosolic ribose-5-phosphate isomerase causes chloroplast dysfunction, late flowering and premature cell death in *Arabidopsis*. Physiol Plant.

[CR41] Yamada Y, Furusawa S, Nagasaka S, Shimomura K, Yamaguchi S, Umehara M (2014). Strigolactone signaling regulates rice leaf senescence in response to a phosphate deficiency. Planta.

[CR42] Yang X, Nian J, Xie Q, Feng J, Zhang F, Jing H, Zhang J, Dong G, Liang Y, Peng J, Wang G, Qian Q, Zuo J (2016). Rice ferredoxin-dependent glutamate synthase regulates nitrogen-carbon metabolomes and is genetically differentiated between *japonica* and *indica* subspecies. Mol Plant.

[CR43] Yang Y, Xu J, Huang L, Leng Y, Dai L, Rao Y, Chen L, Wang Y, Tu Z, Hu J, Ren D, Zhang G, Zhu L, Guo L, Qian Q, Zeng D (2016). *PGL,* encoding chlorophyllide aoxygenase 1, impacts leaf senescence and indirectly affects grain yield and quality in rice. J Exp Bot.

[CR44] You C, Zhu H, Xu B, Huang W, Wang S, Ding Y, Liu Z, Li G, Chen L, Ding C, Tang S (2016). Effect of removing superior spikelets on grain filling of inferior spikelets in rice. Frontiers Plant Sci.

[CR45] Young MD, Wakefield MJ, Smyth GK, Oshlack A (2010). Gene ontology analysis for RNA-seq: accounting for selection bias. Genome Biol.

[CR46] Yu K, Wei J, Ma Q, Yu D, Li J (2009). Senescence of aerial parts is impeded by exogenous gibberellic acid in herbaceous perennial *Paris polyphylla*. J Plant Physiol.

[CR47] Zhao Y, Chan Z, Gao J, Xing L, Cao M, Yu C, Hu Y, You J, Shi H, Zhu Y, Gong Y, Mu Z, Wang H, Deng X, Wang P, Bressan RA, Zhu JK (2016). ABA receptor PYL9 promotes drought resistance and leaf senescence. Proc Natl Acad Sci U S A.

[CR48] Zhou B, Guo Z (2009). Calcium is involved in the abscisic acid-induced ascorbate peroxidase, superoxide dismutase and chilling resistance in *Stylosanthes guianensis*. Biol Plant.

