# Supplementary material for: A 3-bp deletion of WLS5 gene leads to weak growth and early leaf senescence in rice
Source: Rice (N Y). 2019 Apr 29;12:26. doi: 10.1186/s12284-019-0288-8 (PMC6488631; doi:10.1186/s12284-019-0288-8)
Supplement: Supplementary file 1 — Figure S1. Histological characterization of leaves in wild-type and wls5 plants. a, b Cross sections of leaves of wild-type (‘93-11’) and wls5. c-f Longitudinal sections of leaves of wild-type and wls5 plants. Scale bar = 50 μm. Figure S2. Statistics of GO enrichment analysis of differential expression genes between wild-type (WT) and wls5 plants. mRNA was purified from total RNA isolated from tillering-stage plants of WT (‘93-11’) and wls5. Figure S3. Statistics of pathway enrichment analysis of differential expression genes between wild-type (WT) and wls5 plants. mRNA was purified from total RNA isolated from tillering-stage plants of WT (‘93-11’) and wls5. Figure S4. The number of differential expression genes involved in different biological processes, cellular components and molecular functions. mRNA was purified from total RNA isolated from tillering-stage plants of WT (‘93-11’) and wls5. (PDF 2035 kb) [file 12284_2019_288_MOESM1_ESM.pdf]

Fig. S1

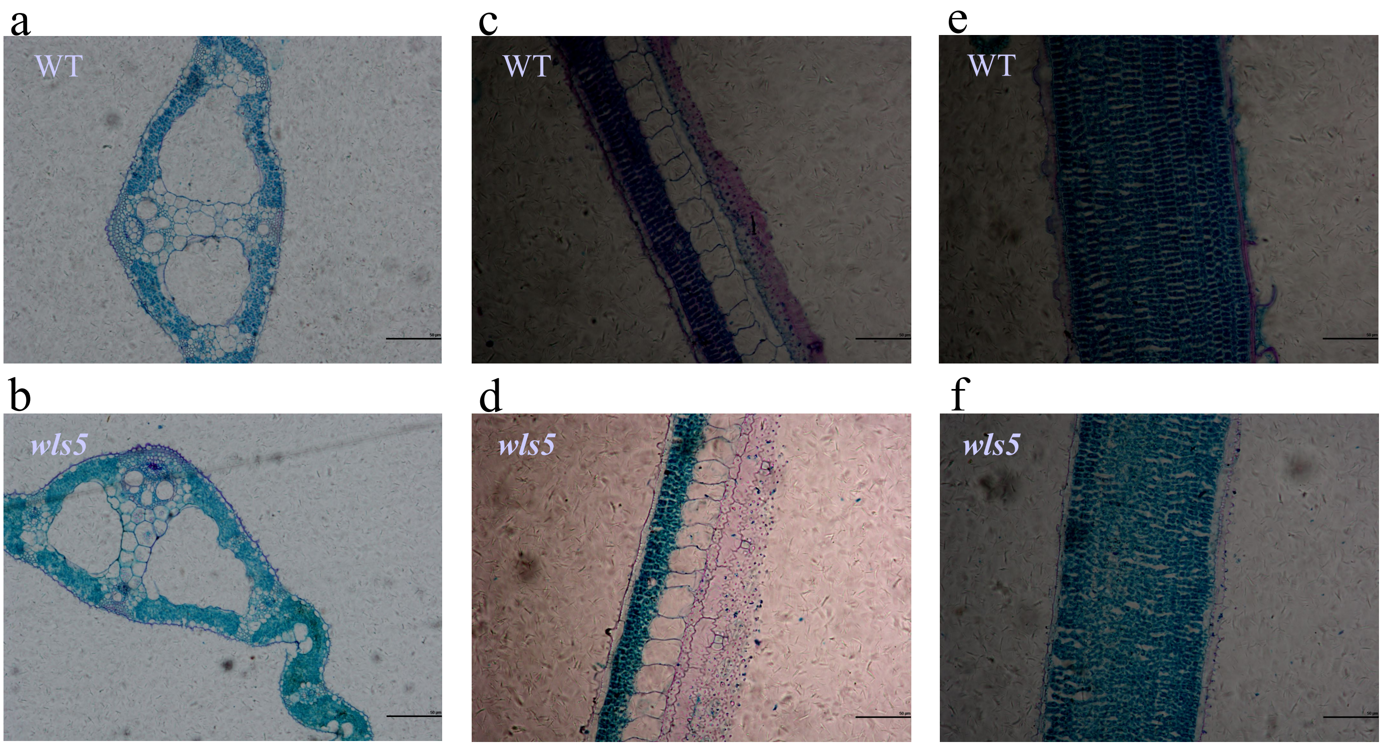

Fig. S1 Histological characterization of leaves in wild-type and *wls5* plants. **a**, **b** Cross sections of leaves of wild-type ('93-11') and *wls5*. **c-f** Longitudinal sections of leaves of wild-type and *wls5* plants. Scale bar = 50 μm.

Fig. S2

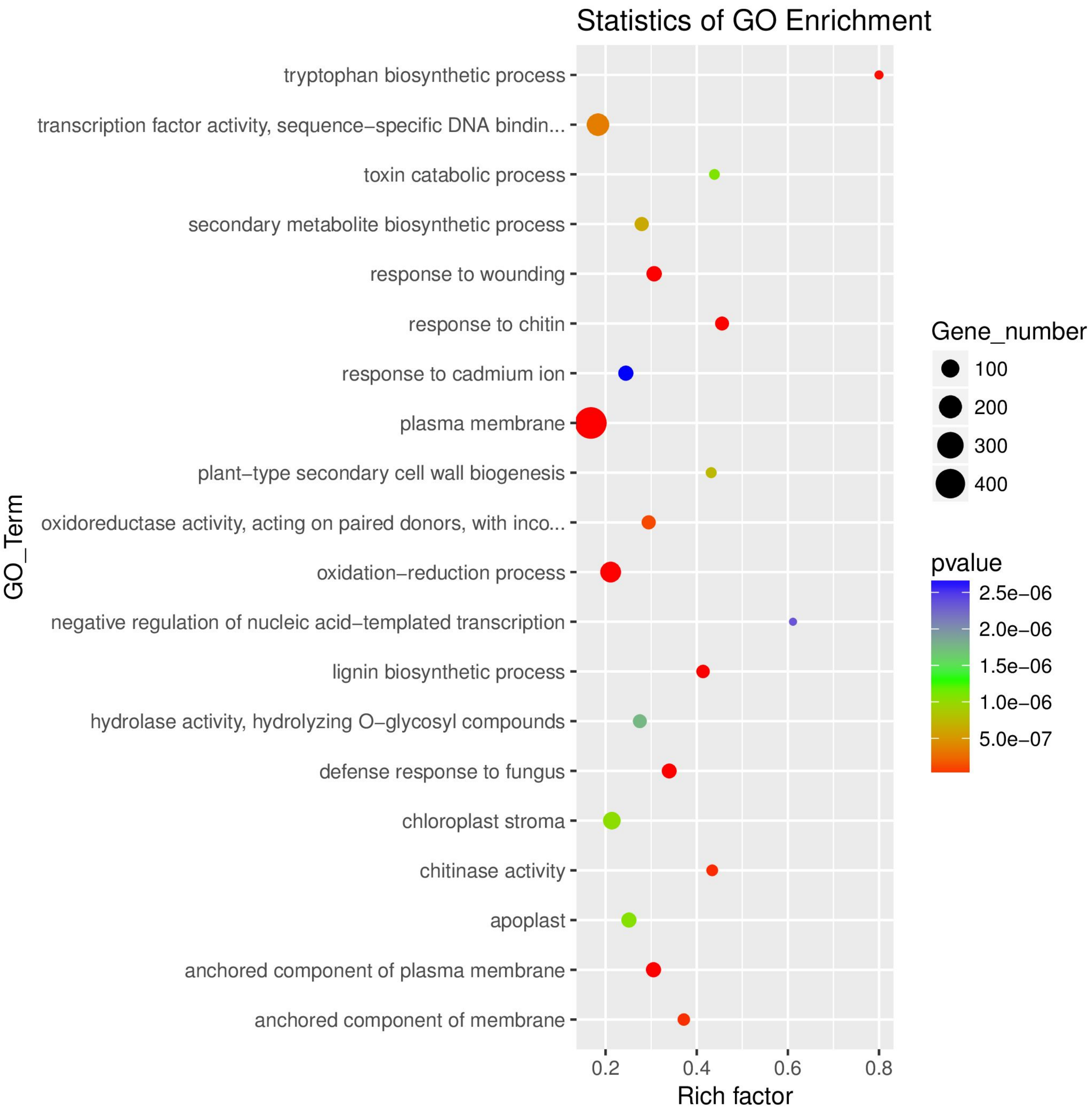

Fig. S2 Statistics of GO enrichment analysis of differential expression genes between wild-type (WT) and *wls5* plants. mRNA was purified from total RNA isolated from tillering-stage plants of WT ('93-11') and *wls5*.

Fig. S3

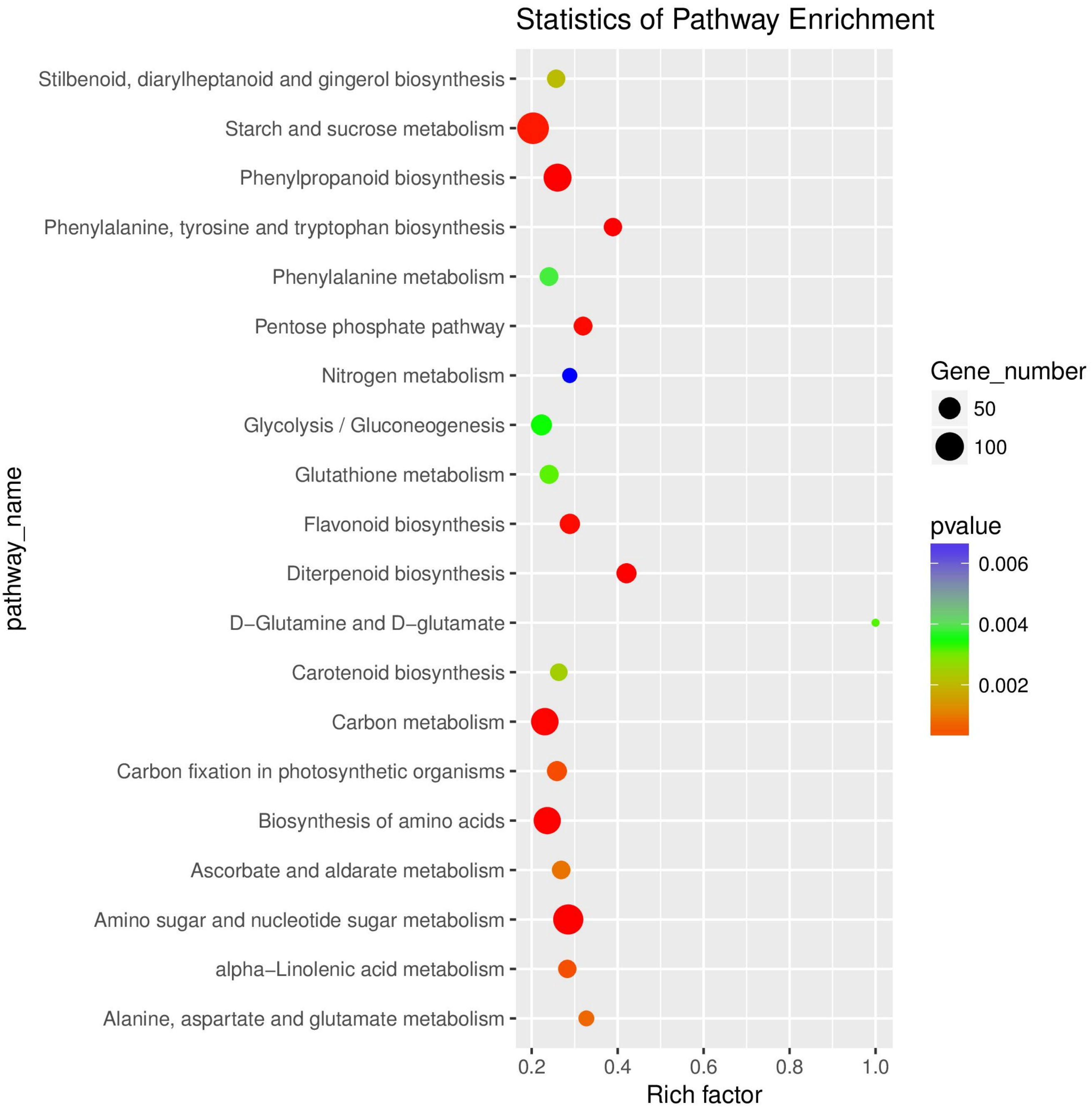

Fig. S3 Statistics of pathway enrichment analysis of differential expression genes between wild-type (WT) and *wls5* plants. mRNA was purified from total RNA isolated from tillering-stage plants of WT (‘93-11’) and *wls5*.

Fig. S4

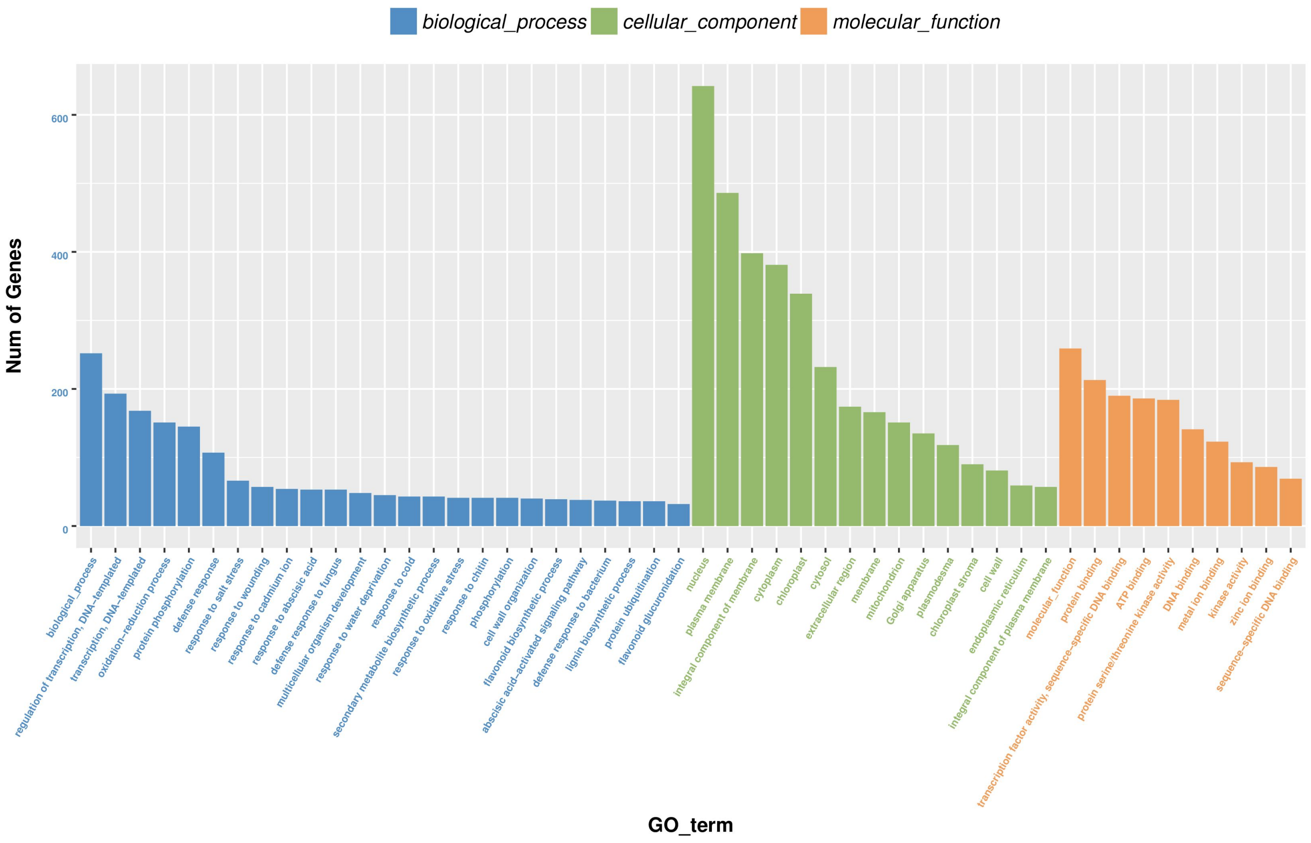

Fig. S4 The number of differential expression genes involved in different biological processes, cellular components and molecular functions. mRNA was purified from total RNA isolated from tillering-stage plants of WT (‘93-11’) and *wls5*.
