# Supplementary material for: A 3-bp deletion of WLS5 gene leads to weak growth and early leaf senescence in rice
Source: Rice (N Y). 2019 Apr 29;12:26. doi: 10.1186/s12284-019-0288-8 (PMC6488631; doi:10.1186/s12284-019-0288-8)
Supplement: Supplementary file 2 — Table S1. The list of primers used in this study (PDF 77 kb) [file 12284_2019_288_MOESM2_ESM.pdf]

Table S1 The list of primers used in this study

Primer name	Primer sequence (5' to 3')	Purpose
M1F	TTGTAACCACCAGCAGCAGGG	InDel marker for fine mapping *WLS5*
M1R	AGCAATGGTACAAATAGCCAAGC	
M2F	ATTTCAGTACAAGGCACCCATGC	InDel marker for fine mapping *WLS5*
M2R	AGGGAGTACCAACTTGCTGTTC	
M3F	TTATGCATGGATTCGACGATC	InDel marker for fine mapping *WLS5*
M3R	GAAAAGCATAGGAGGAGCATT	
M4F	TCCAACATGGCAAGAGAGAG	InDel marker for fine mapping *WLS5*
M4R	TATCACATTCGATTCCAGCATG	
M5F	TTTTCTTCCCCATCCCTCTTTA	InDel marker for fine mapping *WLS5*
M5R	CCTCCTCTAGTAACCTACGTG	
M6F	TCGGGACCATAGCAAGTC	InDel marker for fine mapping *WLS5*
M6R	GCTGTCTTGCGATGACTC	
M7F	TGAAATGTCAGTTTAGCCGG	InDel marker for fine mapping *WLS5*
M7R	GGCCGTTTGAAGAGTACAATA	
M8F	AGATTTTAAGGACGTTCGCTT	InDel marker for fine mapping *WLS5*
M8R	CGGCAATTAGGAGTATGATCG	
M9F	TTGCTGTCTTGCTCATTACC	InDel marker for fine mapping *WLS5*
M9R	AAGAGGACGATGTGCCAA	
M10F	TGCAGACATAGAGAAGGAAGTG	InDel marker for fine mapping *WLS5*
M10R	AGCAACAGCACAACTTGATG	
qUBQ F	AACCAGCTGAGGCCCAAGA	Real time RT-PCR of *UBQ5*
qUBQ R	ACGATTGATTTAACCAGTCCATGA	
qSGR F	GCAATGTCGCCAAATGACG	Real time RT-PCR of *SGR*
qSGR R	GCTCACCACACTCATTCCCTAAAG	
qOsh36 F	CCTGGTGATCTGAAGGTTGT	Real time RT-PCR of *Osh36*
qOsh36 R	CATGGCAACCAGTGTAAAGC	
qRCCR1 F	GGATCGACGATTGATTTCATG	Real time RT-PCR of *RCCR1*
qRCCR1 R	GTCGAGGCGTTCAGAAAGAT	
qOsl57 F	ACCCTAAAGTAAATGAAGTC	Real time RT-PCR of *Osl57*
qOsl57 R	CCTGCTCTTGTCTTGTTA	
qCAO1 F	TTGGCTCAGTTAATGAGGGCAGAATCC	Real time RT-PCR of *CAO1*
qCAO1 R	GGATGCGCACGTTGAGCATCTTTGTGG	
qCHLH F	AACTGGATGAGCCAGAAGAGA	Real time RT-PCR of *CHLH*
qCHLH R	AAATGCAAAAGACTTGCGACT	

qDVR F	AGCCCAGGTTCATCAAGGT	Real time RT-PCR of *DVR*
qDVR R	TGATCACCCTCTCGAAGAACT	
qOsPORA F	ATGGCTCTCCAAGTTCAG	Real time RT-PCR of *OsPORA*
qOsPORA R	TGGCTCACGCTAAGGAAC	
qOsPORB F	CCGCAAGGAGGGAGCGGTG	Real time RT-PCR of *OsPORB*
qOsPORB R	CCCTCTTGGTGCTAAGGCCG	
qLchP2 F	GAAGAAGATCAAGAACGGCC	Real time RT-PCR of *LchP2*
qLchP2 R	TTGCCGGGGACGAAGTTGGT	
qPsbA F	AGAGACGCGAAAGTACAAGC	Real time RT-PCR of *Psb*
qPsbA R	AAGTTGCGGTCAATAAGGTA	
qRpoC1 F	TCCGTCGGAACAACAATCTTG	Real time RT-PCR of *RpoC1*
qRpoC1 R	TCCACGGCTTCTTGTACCAAT	
qRpoC2 F	ATGCATCGCAGGTACACCAA	Real time RT-PCR of *RpoC2*
qRpoC2 R	CCCTCGCGTAAATTGCTTTG	
qRps15 F	AGATACGGAGACTTGCTTCA	Real time RT-PCR of *Rps15*
qRps15 R	GCTCCCTAATATCCAACTGACT	
qV1 F	AGAATCAGCGCGAGAAGAGAACCT	Real time RT-PCR of *V1*
qV1 R	TACACCAGCTTTGGAGGAGCTGAA	
qV2 F	AGCAGATCCGTGATTACATGGCGA	Real time RT-PCR of *V2*
qV2 R	TGCCTCTTCACTCTCTGCAACCAA	
qV3 F	AACGAGAGATCTGGGCTGAATGCT	Real time RT-PCR of *V3*
qV3 R	AGCATTCAGCCCAGATCTCTCGTT	
qAOX1a F	CTTCGCATCGGACATCCATTA	Real time RT-PCR of *AOX1a*
qAOX1a R	TCCTCGGCAGTAGACAAACATC	
qAOX1b F	CCTGCTCAGTTCATCACCATCA	Real time RT-PCR of *AOX1b*
qAOX1b R	GCATAAAACGGAGTGACAATAGC	
qAPX1 F	AGGTGCCACAAGGAAAGATCTGGT	Real time RT-PCR of *APX1*
qAPX1 R	TCAGCAGGGCTTTGTCACTAGGAA	
qAPX2 F	TGGGAAGATGCCACAAGGAGAGAT	Real time RT-PCR of *APX2*
qAPX2 R	TCCGCAGCATATTTCTCCACCAGT	
qcatA F	CAACCGCAACGTCGACAACTTCTT	Real time RT-PCR of *catA*
qcatA R	TTCACCGGCAGCATCAGGTAGTTT	
qcatB F	GCTTGCTTTCTGCCCAGCGATAAT	Real time RT-PCR of *catB*
qcatB R	AAATAGTTTGGGCCAAGACGGTGC	
qOsPOD1 F	ACGTCGGGGTCGCCAACAAC	Real time RT-PCR of *OsPOD1*
qOsPOD1 R	CGAACTCGTCCACCGACGCC	

qSODA1 F	ATCTGGATGGGTGTGGCTAGCTTT	Real time RT-PCR of *SODA1*
qSODA1 R	AGTACGCATGCTCCCAGACATCAA	
qSODB F	TCCGCCGTATAAACTTGATGCCCT	Real time RT-PCR of *SODB*
qSODB R	TGGGTTGCCGTTGTTGTATGCTTC	
wls5g++	ggcaGATGGTGGCCAGCGTGCCGA	pC1300-UBI:Cas9 vector construction, genome editing
wls5 g--	aaacTCGGCACGCTGGCCACCATC	
